# Soil Stoichiometry-Regulated Microbial Carbon Use Efficiency Between Rhizosphere and Bulk Soils in the Temperate Forests of Northeastern China

**DOI:** 10.3390/plants15040652

**Published:** 2026-02-20

**Authors:** Beixing Duan, Ruihan Xiao

**Affiliations:** 1 School of Hydraulic and Electric Power, Heilongjiang University, Harbin 150080, China; duanbx@hlju.edu.cn; 2 Post-doctoral Mobile Research Station of Ecology, Heilongjiang University, Harbin 150080, China; 3 International Joint Laboratory of Hydrology and Hydraulic Engineering in Cold Regions of Heilongjiang Province, Harbin 150080, China

**Keywords:** ecological stoichiometry, CUE, stoichiometry imbalance, rhizodeposition, soil microbial community

## Abstract

In forest ecosystems, rhizodeposition can lead to significant differences in the availability of soil carbon (C), nitrogen (N), and phosphorus (P) between rhizosphere and bulk soils. Soil stoichiometry affects microbial and enzyme nutrient content and determines the abundance and composition of microbes and thus regulates microbial carbon use efficiency (CUE). However, how soil stoichiometry—particularly its variation between the rhizosphere and bulk soil—regulates microbial CUE by shaping microbial biomass, extracellular enzyme stoichiometry, and community composition remains insufficiently quantified. Here, through the C:N, C:P, and N:P ratios for available soil nutrients, microbial biomass, and extracellular enzyme activities—(β-1,4-glucosidase (BG), β-1,4-N-acetylglucosaminodase (NAG), leucine aminopeptidase (LAP), and acid phosphatase (ACP))—and the composition and activity of microbial communities (based on sequencing of bacterial 16S rRNA and fungal ITS genes) in the rhizosphere and bulk soils of five temperate forest ecosystems in northeastern China, we aimed to unravel their integrated effects on microbial CUE. Results indicated that soil C, N, and P and their stoichiometry, microbial community composition, and microbial CUE were significantly different between rhizosphere and bulk soils among all tree species. The disproportionate variation in soil nutrient pools between the rhizosphere and non-rhizosphere regions has led to a stoichiometric imbalance. There was higher microbial CUE in the rhizosphere soil than that in the bulk soil among all tree species. However, the effect pathways of tree species on microbial CUE in the rhizosphere and bulk soils differed. The structural equation model (SEM) further suggested that tree species affected microbial CUE through distinct pathways in different soil compartments. In the rhizosphere, the effect was directly driven by available nutrient stoichiometry. In bulk soil, it was jointly mediated by both available nutrients and microbial biomass stoichiometry. These findings demonstrate that root rhizodeposition shapes microbial carbon cycling by altering soil stoichiometric imbalances, which can strengthen the current understanding of plant–microbe–soil interactions in temperate forests.

## 1. Introduction

Soil is the largest carbon (C) pool (∼1500 Pg C) within the terrestrial ecosystem and plays a key role in regulating global carbon cycling and sequestration. The rhizosphere is one of the most highly active parts of the soil ecosystems, shaped by the interactions between plant roots and soil microorganisms [1,2]. Rhizosphere effects play fundamental roles in regulating soil C and nutrient cycling by mediating soil–plant nutrient transfer [3,4,5]. Rhizodeposition induces distinct physicochemical properties and biological processes between rhizosphere soil (RS) and bulk soil (BS) [6], which may drive differences in C cycling processes [7]. Soil microbial community play a vital role in regulating soil C decomposition and storage. Microbial carbon use efficiency (CUE) (calculation formula is provided in Section 4) serves as a pivotal indicator for characterizing microbial physiological metabolism [8,9], reflecting the capacity for carbon storage in ecosystems [10]. It has been demonstrated that representing microbial CUE as a variable parameter in soil biogeochemical models can significantly improve the accuracy of projections regarding global soil carbon dynamics [11]. Moreover, various tree species differ in soil rhizodeposition due to their unique root exudate compositions and microbial community structures [12]. Therefore, exploring the pathways through which the rhizosphere affects microbial CUE is essential to further understand the mechanisms underlying microbial-driven C cycling in forest ecosystems.

Ecological stoichiometry serves as a powerful tool for elucidating carbon and nutrient cycling pathways within terrestrial ecosystems, uncovering dynamic equilibria underlying biogeochemical processes. According to the prediction of ecological stoichiometric theory, microbial communities modulate their CUE in response to elemental ratios (including both microbial biomass and resource C:N:P ratios) to maintain stoichiometric homeostasis within physiological constraints [13]. For instance, substrate stoichiometry is recognized to have a significant effect on microbial CUE by regulating microbial investment in extracellular enzymes for resource acquisition [14,15]. However, Cao et al. [16] suggested that modifications in enzymatic stoichiometry served as a fundamental strategy for microorganisms to maintain metabolic homeostasis under changing resource efficiency conditions [16]. Previous studies have also reported that microbial biomass stoichiometry is a key predictor of soil microbial CUE across different tree species [17]. Meanwhile, the interactions among soil substrate, microbial biomass, and enzymatic activity on microbial CUE should also be considered [18]. In forest ecosystems, the significant differences in soil stoichiometry among soil compartments [19] and plant species [20] could lead to different microbial CUE. However, the factors that determine the strength and direction of these pathways, particularly the role of tree species in shaping them, are poorly understood. Therefore, further research is necessary to clarify the characteristics and mechanisms of soil stoichiometry affecting microbial CUE between rhizosphere and bulk soils.

The soil microbial activity and community composition also play critical roles in regulating soil C cycling in both rhizosphere and bulk soils [21]. Bacterial and fungal communities demonstrate distinct soil carbon substrate preferences and different CUE [22,23]. Previous studies have indicated that the variation in microbial CUE is closely related to bacterial community, showing a positive relationship with bacterial diversity but no significant correlation with fungal diversity [24,25]. However, the negative or neutral correlations between microbial diversity and CUE were also reported [26,27]. Moreover, a lower fungi-to-bacteria ratio is associated with a reduced ability to utilize recalcitrant C, potentially leading to lower microbial CUE [28]. Plant species drive the difference in microbial activity and community composition between rhizosphere and bulk soils primarily through root-mediated alterations in soil resource stoichiometry [29,30]. Changes in soil microbial community composition may lead to differences in microbial CUE between rhizosphere and bulk soils. Therefore, understanding the response of microbial properties to rhizodeposition and their influence on microbial CUE is critically important.

Temperate forests in northeastern China account for more than one-third of the total national forest land area and are characterized by distinctive species composition and high biodiversity [31]. It plays a central role in the national C balance in China owing to its large, forested area and aboveground biomass. Previous studies have suggested that various tree species can lead to significant differences in soil properties [32,33]. However, how these tree species-induced differences in soil properties via rhizosphere effects regulate microbial CUE remains largely unexplored. Thus, the aim of our study is to reveal the rhizosphere effects of different tree species on soil stoichiometry and microbial community composition, and how these responses affect microbial CUE. Specifically, we selected five tree species representative of the temperate forest ecosystems (representing the dominate tree species), i.e., the *Betula platyphylla* Suk., the *Fraxinus mandschurica* Rupr., the *Populus davidiana* Dode., the *Larix gmelinii* (Rupr.) Kuzen., and the *Pinus koraiensis Siebold et* Zucc. Soil stoichiometry characteristics (available nutrient C:N:P ratios, ectoenzyme activity C:N:P ratios, and microbial biomass C:N:P ratios), microbial activity, and bacterial and fungal community composition were measured in both the rhizosphere and bulk soils among five tree species in the temperate forests of the northeast China. We hypothesized that: (1) compared to bulk soil, there would be a significantly higher microbial CUE in rhizosphere soil due to the C and nutrients input from the plant roots; and (2) the effects of tree species on soil microbial composition, stoichiometry, and microbial CUE differ between rhizosphere and bulk soils due to root systems and their corresponding physiological processes.

## 2. Results

### 2.1. Soil C, N, P, Available Nutrients, Microbial Biomass, and Enzymatic Activities

The rhizosphere soil available nutrients, microbial biomass, enzymatic activity, and total nutrients were all significantly higher than those in the bulk soil in all tree species (*p* < 0.05) (Table 1). In the rhizosphere soil, the DOC, AN, MBC, MBP, and NAG + LAP concentrations were the highest in *B. platyphylla*. The MBN and BG concentrations were higher in the rhizosphere soil of *P.davidiana*. The AP, ACP, SOC, TN, and TP concentrations were the highest in the rhizosphere soil of *P. koraiensis*. In the bulk soil, the DOC, AN, AP, ACP, SOC, TN and TP concentrations were the highest in P. koraiensis. The MBC, MBN, MBP, BG, and NAG + LAP concentrations were the highest in the bulk soil of *B. platyphylla*.

### 2.2. Soil Stoichiometry and Stoichiometric Imbalances

**Figure 1:**
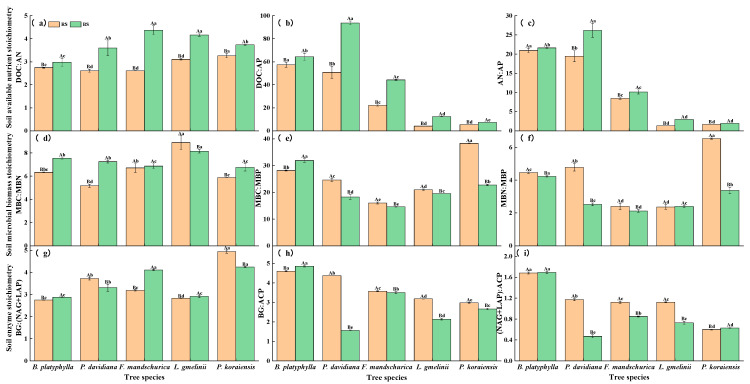
Soil available nutrient stoichiometry, microbial biomass stoichiometry, and enzyme stoichiometry of rhizosphere (RS) and bulk soil (BS) in different tree species. Soil DOC:AN (**a**), DOC:AP (**b**), AN:AP (**c**), MBC:MBN (**d**), MBC:MBP (**e**), MBN:MBP (**f**), BG:(NAG + LAP) (**g**), BG:ACP (**h**), and (NAG + LAP):ACP (**i**). Note: Different uppercase letters indicate significant differences between the rhizosphere and bulk soil (*p* < 0.05); different lowercase letters indicate significant differences among the tree species (*p* < 0.05).

There were significant differences in the soil available nutrient stoichiometry, microbial biomass stoichiometry, and enzyme stoichiometry in the rhizosphere and bulk soil among different tree species (*p* < 0.05) (Figure 1). The two-way ANOVAs indicated that rhizodeposition, tree species and their interaction had significant effects on DOC:AP, AP:AN, MBC:MBN, MBC:MBP, MBN:MBP, BG:(NAG + LAP), and (NAG + LAP):ACP (*p* < 0.001, Table S1). Compared with the rhizosphere soil, there were significantly higher DOC:AN, DOC:AP, AP:AN, and MBC:MBN values in the bulk soil (*p* < 0.05). However, the MBC:MBP, MBN:MBP, BG:ACP and (NAG + LAP):ACP values were significantly higher in the rhizosphere soil (*p* < 0.05).

**Figure 2:**
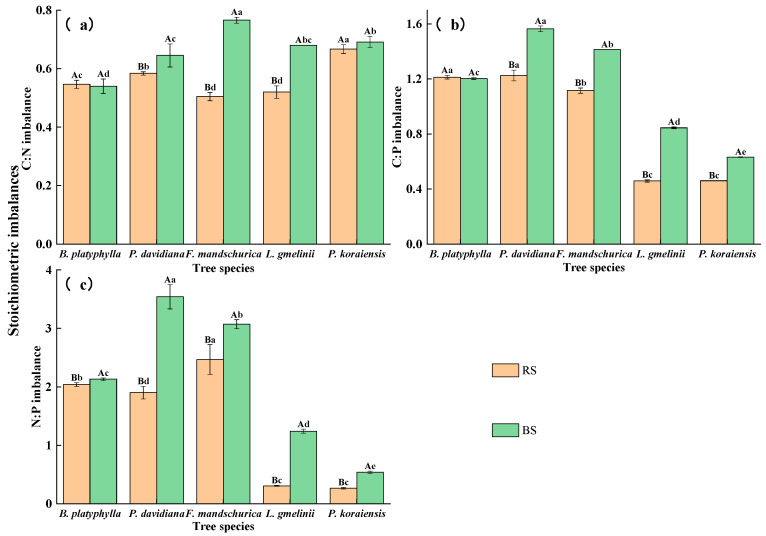
Stoichiometric imbalances between soil microbial communities and their resources in the rhizosphere (RS) and bulk soil (BS) among five tree species. C:N imbalance (**a**), C:P imbalance (**b**), and N:P imbalances (**c**).

The soil stoichiometric imbalances of C:N, C:P, and N:P ranged from 0.50 to 0.77, from 0.46 to 1.56, and from 0.26 to 3.54, respectively (Figure 2). The C:N imbalance, C:P imbalance, and N:P imbalances in the bulk soil were significantly higher than those in the rhizosphere soil (*p* < 0.05). The rhizodeposition, tree species, and their interaction had significant effects on the stoichiometric imbalances (*p* < 0.001, Table S1).

### 2.3. TERs and Microbial Carbon Use Efficiency

**Figure 3:**
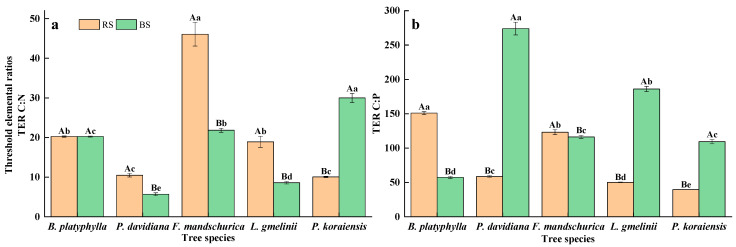
Changes in the threshold elemental ratios (TERs) of soil microbial communities in rhizosphere (RS) and bulk soil (BS) among five tree species. TERC:N (**a**), TERC:P (**b**).

There were significant differences in TERs between the rhizosphere and bulk soil among tree species (*p* < 0.05) (Figure 3). Across most tree species (*B. platyphylla*, *P. davidiana*, *F. mandschurica*, and *L. gmelinii*), the TER C:N ratio was significantly elevated in the rhizosphere relative to bulk soil. In contrast, *P. koraiensis* exhibited a significantly higher ratio in bulk soil (*p* < 0.05). The TER_C:P_ in the rhizosphere soil of *B. platyphylla* and *F. mandschurica* was greater than that in the bulk soil, while that in the bulk soil of *P. davidiana*, *L. gmelinii*, and *P. koraiensis* was greater than that in the rhizosphere soil (*p* < 0.05).

The microbial CUE values were 0.2–0.43 and 0.18–0.35 in rhizosphere and bulk soil, respectively, among five tree species (Table 2). The rhizodeposition, tree species, and their interaction had significant effects on the stoichiometric imbalances (*p* < 0.005, Table 1). Furthermore, the microbial CUE differed greatly among the five tree species. In both rhizosphere and bulk soils, the maximum and minimum microbial CUE values were found in *L. gmelinii* and *P. davidiana*, respectively.

### 2.4. Soil Microbial Community Composition

**Figure 4:**
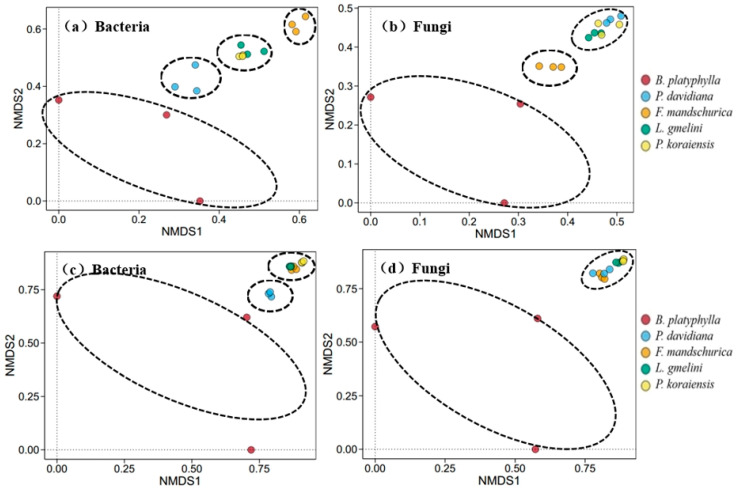
Non-metric multidimensional scaling (NMDS) of soil bacterial and fungal communities based on Bray–Curtis distances among different tree species (plots (**a**,**b**) represent soil in rhizosphere and (**c**,**d**) in bulk).

Shannon indices indicated significantly higher bacterial and fungal alpha diversity in rhizosphere soil than bulk soil among all tree species (*p* < 0.05; Table 3). Meanwhile, alpha diversity also differed significantly among the five tree species in both soil compartments. Soil bacterial and fungal beta diversity also varied significantly in both rhizosphere and bulk soils among five tree species (Figure 4). The distribution of soil microbial communities in *B. platyphylla* was clearly separated from other tree species in both rhizosphere and bulk soils. Meanwhile, soil bacterial communities in *P. davidiana* tended to group together in both soil compartments (Figure 4a,c). In comparison, it only tended to group together in *F. mandschurica* in rhizosphere soil (Figure 4a). Soil fungal communities also tended to group together in *F. mandschurica* in rhizosphere soil (Figure 4b). In bulk soil, fungal communities in the other four tree species (except *B. platyphylla*) were difficult to separate (Figure 4d).

**Figure 5:**
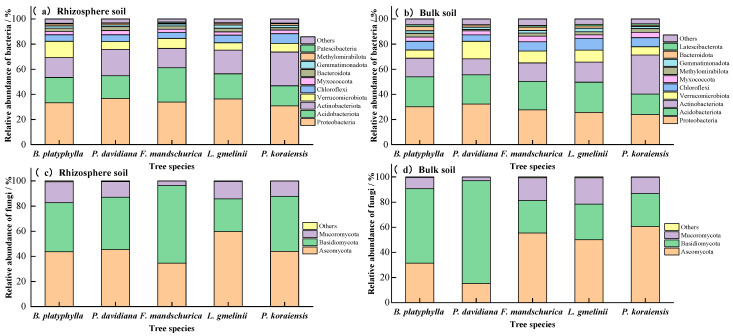
Relative abundances of soil bacterial and fungal community compositions at the phylum level, depicting only groups exceeding 1% relative abundance; all others are categorized as “Other” (plots (**a**,**c**) represent soil in rhizosphere and (**b**,**d**) in bulk).

The soil bacterial community was dominated by the phyla *Proteobacteria*, *Acidobacteria*, *Actinobacteria*, *Verrucomicrobia*, and *Chloroflexi* across all tree species and soil compartments (Figure 5). In the rhizosphere soil, the abundance of *Actinobacteriota* and *Chloroflexi* was highest in *P. koraiensis*, and the lowest in *F. mandschurica* (*p <* 0.05). The abundance of *Proteobacteria*, *Acidobacteriota*, and *Verrucomicrobiota* was similar among all five tree species. In both soil compartments, *Ascomycota*, *Basidiomycota*, and *Mucoromycota* were the dominant fungal communities at the phyla level among all the tree species. In the rhizosphere soil, the relative abundance of Ascomycota was highest in *L. gmelini*, the lowest in *F. mandschurica* (*p <* 0.05). The abundance of *Basidiomycota* was highest in *F. mandschurica*, the lowest in *L. gmelini* (*p* < 0.05). The abundance of *Mucoromycota* was highest in *B. platyphylla*, but the lowest in *F. mandschurica* (*p <* 0.05).

### 2.5. Effects of Soil Stoichiometry on Microbial Properties

**Figure 6:**
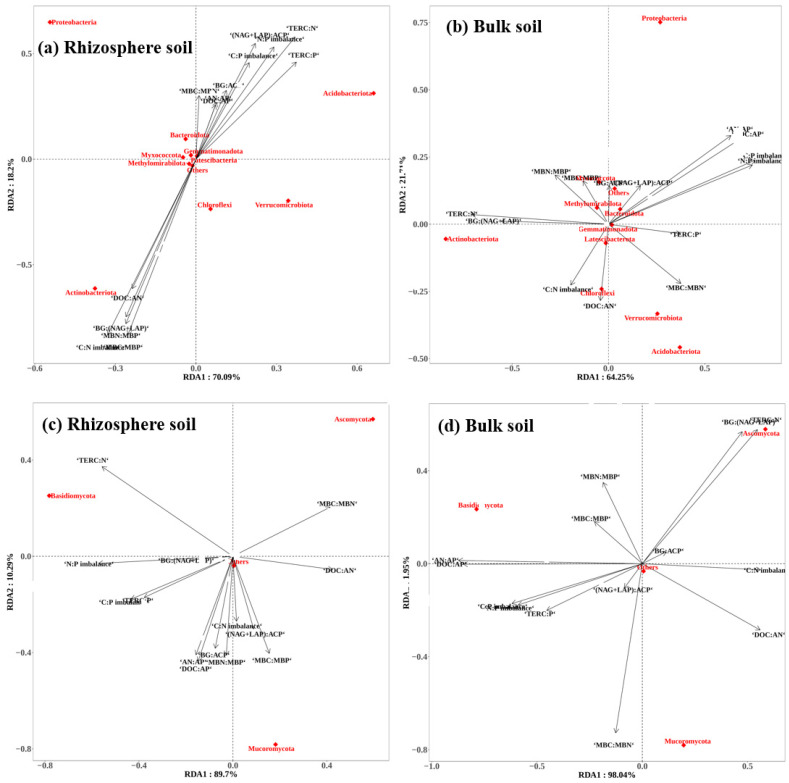
RDA of the abundant bacterial and fungal communities at the phylum level and soil stoichiometries for the samples from five tree species in rhizosphere (**a**,**c**) and bulk (**b**,**d**) soil.

The results of RDA suggested that soil stoichiometry explained 88.29–99.99% of the variation in microbial community compositions (first two axes) in rhizosphere soils (Figure 6). There were close relationships between bacterial community compositions and soil DOC:AN, MBC:MBN, MBN:MBP, BG:(NAG + LAP), (NAG + LAP):ACP, C:N imbalance, N:P imbalance, TER_C:N_, and TER_C:P_ (*p* < 0.05). TER_C:N_ were significantly related to the fungal community compositions (*p* < 0.05). In the bulk soil, the cumulative contribution of variances of microbial communities to soil stoichiometries was 85.96–99.99% for the first two axes. The RDA showed that soil DOC:AP, AN:AP, BG:(NAG + LAP), C:N imbalance, N:P imbalance, TER_C:N_ and microbial CUE were closely related to the bacterial community compositions (*p* < 0.05).The DOC:AN, DOC:AP, AN:AP, MBC:MBN, C:P imbalance, N:P imbalance, and TER_C:N_ were closely related to the fungal community compositions (*p* < 0.05).

### 2.6. Responses of Soil Stoichiometry and Microbial Composition to CUE

**Figure 7:**
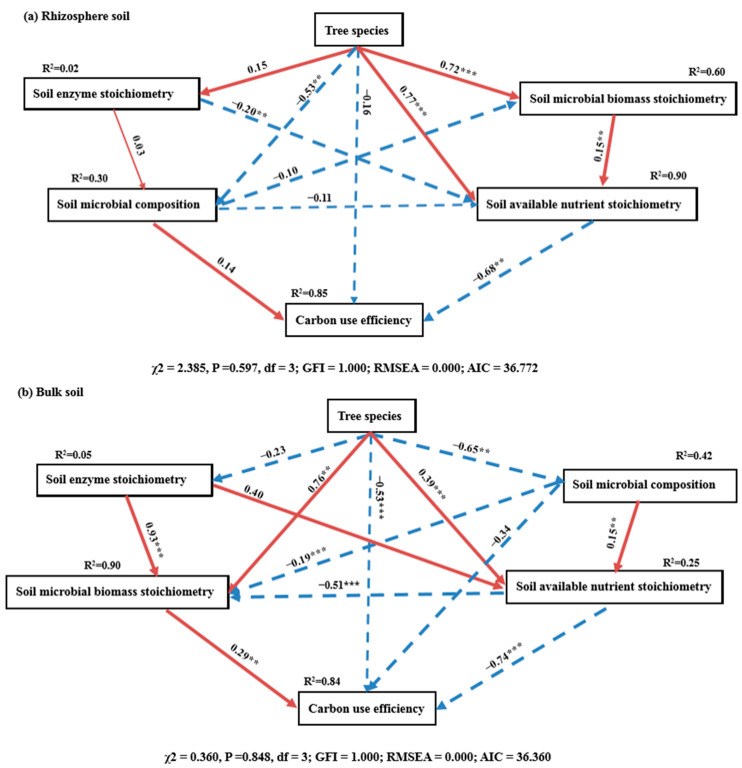
SEM depicting the differential regulatory pathways of tree species on microbial carbon use efficiency (CUE) in rhizosphere (**a**) and bulk (**b**) soils. Notes: Red solid arrows indicate positive effects; blue dotted arrows represent negative effects. R2 values represent the proportion of variance explained for each variable. The values beside the arrow represent standardized coefficient. Significance of the correlation is indicated at the 0.001 (***) and 0.01 (**) level.

The SEM supported the relationships among tree species, soil stoichiometry, microbial composition, and soil microbial CUE (Figure 7), which explained 85% and 84% of the variation in soil microbial CUE in the rhizosphere and bulk soils, respectively. Specifically, in the rhizosphere soil, tree species had significantly direct effects on soil microbial biomass stoichiometry, available nutrient stoichiometry, and microbial composition. This result showed that soil available nutrient stoichiometry was the main controller of microbial CUE in the rhizosphere soil. Meanwhile, in the bulk soil, tree species had significantly direct effects on soil microbial biomass stoichiometry, available nutrient stoichiometry, microbial composition, and microbial CUE. Soil available nutrient stoichiometry and microbial biomass stoichiometry played vital roles in regulating microbial CUE.

## 3. Discussion

Our study showed that both rhizodeposition and tree species significantly influenced the soil stoichiometry, microbial CUE, and microbial community (Figure 1, Table 1, Table 2 and Table 3). The revised text now reads: In terrestrial ecosystems, plants release C (e.g., labile carbon) through root exudates into the rhizosphere, which stimulates microbial activity and primes the mineralization of soil organic matter—a biochemical process whereby microbes convert organic nitrogen and phosphorus into plant-available mineral forms [1,34,35]. In addition, the rhizosphere soil, being the most active microbe habitat, has very high ecological enzyme activity [20,36]. Hence, soil available nutrients, microbial biomass, and enzymatic activity in the rhizosphere were much richer than those in bulk soils (Table 1). These results could lead to differences in soil available nutrient stoichiometry, microbial stoichiometry, and enzyme stoichiometry between rhizosphere and bulk soils. Previous studies have reported that the microbial community compositions in the rhizosphere soil could be extremely sensitive to alterations in soil nutrient availability and stoichiometry (e.g., C, N, P substrates) [37,38,39]. Our results further revealed that the differences in soil nutrient availability and bacterial and fungal community composition and diversity result in different microbial nutrient use efficiency between the rhizosphere and bulk soil. Moreover, studies have documented that tree species, through the specific chemical composition of their root exudates (e.g., differing ratios of sugars, organic acids, and phenolic compounds), could affect soil nutrient availability, extracellular enzyme activities (e.g., amino acid-related enzymes), and bacterial and fungal community composition in both rhizosphere and bulk soils [5,12]. Changes in the allocation of belowground C by tree species can alter organic matter decomposition, ultimately impacting soil microbial CUE.

The stoichiometric relationship in soil is a critical driver of carbon and nutrient cycling as it directly reflects microbial resource demands [40]. Partially consistent with our first hypothesis, not all soil stoichiometric ratios in the rhizosphere soil were significantly higher than that in the bulk soil. Our results revealed that there were higher available nutrients, microbial biomass, and enzymatic activity in the rhizosphere soil than in the bulk soil (Table 1). Meanwhile, the soil DOC:AN, DOC:AP, AN:AP, and MBC:MBN in the rhizosphere were significantly lower than those in the bulk soil (*p* < 0.05), indicating a greater demand for rhizosphere microorganisms for carbon resources. Meanwhile, the higher MBC:MBP and MBN:MBP in the rhizosphere soil also indicated a microbial nutrient mismatch relative to soil resource availability. Based on the high turnover rate of soil available nutrients and soil microbial biomass, the stoichiometric imbalance can be better determined. Unlike previous studies that used the soil total nutrient content to calculate stoichiometric imbalances [41], our research indicated that the soil available nutrient stoichiometry in rhizosphere and bulk soils showed weak or no stoichiometric homeostasis among all tree species. The logarithmic scaling ratios of C-, N-, and P-acquiring enzyme activities in our study (based on BG, NAG + LAP, and ACP, respectively) approximated 1:1:1, consistent with global and regional averages for C:N:P acquisition [42]. However, significant differences in enzyme stoichiometry were observed in both the rhizosphere and bulk soils among different tree species (Figure 1). This result is consistent with previous studies, which demonstrated that vegetation type, rather than macro-climate, is the primary driver of soil enzyme activities and stoichiometry in subalpine forests [43], indicating the major roles of tree species in regulating soil enzyme activities and stoichiometry.

Microbial CUE is recognized as a vital indicator of soil carbon sequestration dynamics, as it directly mediates microbial biomass carbon retention while reducing system-level carbon losses [44,45]. In our study, microbial CUE was from 0.16 to 0.43, which is similar to the CUE from the broad range of ecosystems (0.27 ± 0.11 based on C:N stoichiometry) [18]. Supporting our first hypothesis, there were higher microbial CUE values in the rhizosphere soil compared with the bulk soil among the five tree species. This phenomenon can be explained by two reasons. First, the lower microbial CUE in the bulk soil may be attributed to the lower nutrient availability and, consequently, elevated nutrient acquisition costs [46,47]. Specifically, under nutrient limitation, microbes prioritize energy allocation for resource acquisition rather than growth, thus reducing microbial CUE. Second, microbial C investment in enzyme production can reduce microbial CUE [48,49]. Yet, higher enzyme activity in rhizosphere soil can compensate for carbon consumption in enzyme synthesis, leading to higher biomass yield and improving microbial CUE. Further, compared with the broad-leaved tree species, the microbial CUE in the coniferous tree species was higher in our study, indicating that soil microorganisms in coniferous forests have a higher utilization efficiency of carbon resources and a greater potential for carbon storage in the soil. This phenomenon may be related to higher P availability and lower litter quality in the coniferous forests in our study (Table 1). Higher P availability could enhance microbial CUE and high-quality litter inputs stimulated microbial activity but reduced microbial CUE [50]. In this study, there was significantly higher P availability in *L. gmelinii*, and *P. koraiensis* than that in other forests (Table 1 and Table 4), which could indicate higher microbial CUE. Meanwhile, compared to the broad-leaved forests, the coniferous forests were generally characterized by low quality litter inputs and slow decomposition. The slow but steady C use could also lead to a higher microbial CUE in coniferous forests in our study. Nevertheless, TERs serve as effective indicators for deciphering microbial metabolic regulation, where shifts between nutrient limitation (enhanced nutrient use efficiency) and energy limitation (elevated CUE) are quantitatively reflected in elemental ratios [20,45]. In this study, the DOC:AN and DOC:AP have lower values in comparison to TER_C:N_ and TER_C:P_. These results suggest a shift in microbial life-history strategy from high nutrient use efficiency (NUE) towards high CUE, indicating that temperate forests are more susceptible to C limitation [8,51].

Our study identified that the soil microbial community diversity varied significantly between rhizosphere and bulk soil, as well as among the tree species (Table 3). This result confirmed our second hypothesis. Both bacterial and fungal alpha diversity were significantly lower in the bulk soil compared to the paired rhizosphere soil, which is similar to previous [52,53]. This is mainly due to the release of nutrients from plant roots into the surrounding soil, which induces a higher nutrient availability for microorganisms growth and ultimately affects the microbial diversity between the root–soil systems [37,54]. There were similar soil microbial community compositions, but the abundance of the dominant microbes was significantly different at the phylum level in rhizosphere and bulk soils across five tree species (Figure 5). The abundance of bacterial *Proteobacteria* in the rhizosphere soil was significant higher than that in the bulk soil among all the tree species (Figure 5), which might be due to higher resource availability compared to bulk soil [37]. However, there was no unified pattern for other important bacterial phyla in rhizosphere and bulk soils for different tree species. In contrast to bacteria, the abundance of the primary fungal communities in the soil (*Ascomycota*, *Basidiomycota*, and *Mucoromycota*) varied significantly among tree species. Due to the different ecological strategies, soil fungi exhibit lower resistance and stability to land use change than soil bacteria [55], which induces that fungal community was more strongly influenced by the tree species.

Theoretical models and controlled experiments have established that substrate quality and microbial community composition are key determinants of microbial CUE [10,18,24,25]. Our research further confirmed this statement. On the one hand, the diversity of soil microbial community directly affected CUE. We found significant positive relationships between microbial CUE and diversity, indicating that increased bacterial and fungal diversity could enhance microbial CUE (Table S2). However, divergent relationships (i.e., positive, neutral, or negative) between them were also reported in previous studies [25,27,56]. This inconsistency is likely from variations in microbial community components among different experiments. Consistently positive correlations between microbial diversity and soil C cycling function were observed only in low diversity communities (≤10 species) [57]. On the other hand, soil microbial community composition can also affect microbial CUE by influencing soil stoichiometry. The RDA showed that the soil available nutrient stoichiometry, microbial stoichiometry, and enzyme stoichiometry were closely linked to the soil microbial communities (Figure 6). These correlations confirm the classical theory that microbial communities modulate their CUE in response to variations in substrate elemental ratios (C:N:P) to maintain stoichiometric homeostasis within physiological constraints [11]. Nevertheless, the interrelationships among soil microorganisms, stoichiometry, and microbial CUE were different between rhizosphere and bulk soils in our study. This result further highlights the role of rhizosphere effects in influencing microbial CUE and soil carbon cycling.

Additionally, consistent with our second hypothesis, the SEM results further supported the effects of tree species on soil microbial composition, stoichiometry, and microbial CUE (Figure 7). In rhizosphere soil, soil microbial CUE was strongly regulated by soil available nutrient stoichiometry, which exhibited significant mediation through microbial biomass stoichiometry and extracellular enzyme stoichiometry. This finding reveals a cascading “resource-microbe-function” regulatory pathway within the rhizosphere micro-domain [58]. Furthermore, the effects of tree species on soil microbial compositions and biomass stoichiometry suggested that the influence of microbial diversity and activity on soil available nutrient stoichiometry was widely distributed among different plant types. Additionally, soil microbes in rhizosphere soil were found to have indirect impact on microbial CUE. Compared to the rhizosphere soil, the bulk soil microbial CUE was more sensitive to the variations in available nutrients and microbial biomass stoichiometry. This phenomenon may be due to the fact that the microbes in the bulk soil acquire nutrients directly from the substrate, which comes from the input of organic matter from plant litter. Taken together, our findings indicated that variations in soil microbial composition and enzyme stoichiometry were potential factors leading to microbial CUE changes, while soil available nutrient stoichiometry and microbial biomass stoichiometry were direct factors affecting microbial CUE. Nevertheless, due to the long-term and complex interactions between plant, soil, and microbes, more field and laboratory studies are necessary to further clarify the role of rhizosphere effects in shaping soil C cycling mechanisms.

In this study, we found significant differences in soil microbial CUE between rhizosphere and bulk soils among five tree species in the northeast, China. Tree species affected microbial CUE mainly by regulating soil available nutrients, microbial biomass stoichiometry, and soil microbial community. However, there are still limitations in our study. First, the only-once-collected field data can introduce inherent limitations. Soil microbial community, nutrients, and plant root traits varied significantly over time, and the interaction between plant–soil–microbe is a long-term and complex process [59]. Therefore, more field studies are necessary to better explore the relationship between soil microbial CUE and its associated factors. Secondly, in this study, the mechanisms of soil microbial community regulating microbial CUE were clarified only at the phylum level. In fact, previous studies have suggested the relationship between soil C cycling and microorganisms at the genus level [60]. Therefore, to better explore the pathway and mechanisms of soil microbial on CUE, more detailed research, including laboratory studies, are needed.

## 4. Materials and Methods

### 4.1. Study Site

This study was carried out in the Xiaoling region of Heilongjiang Province in Northeast China (127°05′–127°34′ E, 45°23′–45°52′ N). The area is characterized by a temperate continental monsoonal climate, with mean annual temperature of 2.0 °C and mean annual precipitation ranging from 550 to 700 mm [61]. The frost-free season duration is approximately 90–110 d, and the snowpack is from mid-November to mid-April. The soils are classified as Alfisols (Eutroboralfs) according to the United States Soil Taxonomy. Dominant tree species include *Pinus koraiensis Siebold et* Zucc., *Larix gmelinii* (Rupr.) Kuzen., *Betula platyphylla* Suk. *Populus davidiana* Dode., and *Fraxinus mandschurica* Rupr.

### 4.2. Experimental Design and Soil Sampling

The field surveys were conducted in July 2023. In this study, five typical forest ecosystems were selected, i.e., *Betula platyphylla* Suk. forest (*B. platyphylla*), *Fraxinus mandschurica* Rupr. forest (*F. mandschurica*), *Populus davidiana* Dode. forest (*P. davidiana*), *Larix gmelinii* (Rupr.) Kuzen. forest (*L. gmelinii*), and *Pinus koraiensis Siebold et* Zucc. forest (*P. koraiensis*). For each forest type, three replicates of 20 m × 20 m plots were set randomly in each forest type (a total of 15 plots), maintaining ≥20 m among plots to ensure spatial independence. The basic information of five forest ecosystems is presented in Table 4.

In each plot, four representative trees were selected based on the average diameter at breast height (DBH) and height for rhizosphere soil collection. Rhizosphere soil samples were collected from all plots. Briefly, for each selected canopy tree, living fine roots (diameter ≤ 2 mm) were carefully excavated from the top 10 cm soil within 1 m radius from the trunk along a south-to-north transect beneath. During the sampling process, we excavated the soil with the complete root system using a shovel (the volume depended on the extent of the root system itself). After gently shaking off the soil that was not associated with root system, we vigorously shook off all the soil attached to the root surface, and quickly placed it in a sterile bag, which was considered to be the rhizosphere soil [62]. Rhizosphere soil samples from each plot were thoroughly mixed to form one composite sample. Within each plot, composite bulk soil samples (0–10 cm depth) were obtained from 15 systematically distributed sampling points following an S-shaped pattern, using a stainless-steel auger (5 cm diameter). After manually removing extraneous materials, each sample was divided into three parts. One part was air-dried at room temperature, sieved (<2 mm), and reserved for physicochemical analysis. The remaining two were stored at −80 °C and 4 °C for microbial biomass and enzymatic activity assays, respectively.

### 4.3. Soil Analysis

#### 4.3.1. Chemical Properties Analysis

Soil water content (SWC) was determined gravimetrically (about 20.0 g fresh soil) by oven-drying the fresh soil at 105 °C for 24 h to constant weight. Soil pH was measured using a pH meter (PHS-3C, Shanghai, China) in a 1:2.5 soil:water suspension. Soil organic carbon (SOC) and total nitrogen (TN) were measured by dry combustion using an elemental C/N analyzer (LECO, St Joseph, MI, USA). Soil total phosphorus (TP) was determined by the molybdenum-blue colorimeter, with extract phosphorus solutions from 0.5 M sodium bicarbonate [63]. Dissolved organic carbon (DOC) and nitrogen (DON) were determined with an elemental TOC analyzer (Multi N/C 3100, Analytik Jena GmbH, Jena, Germany) after extracting soil solution using 0.5 mol L^−1^ K_2_SO_4_. Soil available nitrogen (AN) and phosphorus (AP) were determined by a continuous flow analyzer (Analytical AA3 Auto Analyzer 3HR, SEALanalysis Inc., Norderstedt, Germany) after extraction with 0.1 mol L^−1^ KCl and 0.5 mol L^−1^ NaHCO_3_, respectively (Table 1).

Soil microbial biomass carbon, nitrogen, and phosphorus (MBC, MBN, and MBP) were quantified by chloroform fumigation–extraction [64]. The concentrations of MBC and MBN were calculated by subtracting the values extracted from fumigated soil samples from those extracted from non-fumigated soil samples, using 0.5 M K_2_SO_4_. The total organic carbon and nitrogen contents in the extracts were measured using an elemental C/N analyzer (LECO, St Joseph, MI, USA). The conversion factors were 0.45 and 0.54, respectively. The concentration of MBP was calculated by subtracting the values extracted from fumigated soil samples from those extracted from non-fumigated soil samples, using 0.5 M NaHCO_3_. The phosphorus contents in the extracts were measured by ultraviolet spectrophotometer (Hitachi UV2300, Hitachi, Tokyo, Japan) at 700 nm. The conversion factor was 0.4 [65].

The potential activities of four soil enzymes, including β-1,4-glucosidase(BG), β-1,4-N-acetylglucosaminidase(NAG), leucine aminopeptidase (LAP), and acid phosphatase(ACP) were quantified by the method of Verchot and Borelli [66] (Table 1). In this method, enzyme activity was evaluated colorimetrically using ρ-nitrophenol substrates. Briefly, fresh soil samples (2.5 g) were combined with 50 mL of 50 mM acetate buffer and shaken at 25 °C for 40 min at 180 rpm in a constant-temperature incubator. Subsequently, 2 mL of the resulting slurry was mixed with an equal volume (2 mL) of enzyme substrate solution. Following incubation, the mixture was centrifuged at 2000 rmp for 5 min. A 1 mL aliquot of the supernatant was then transferred to 0.2 mL of 1 mol L^−1^ NaOH and diluted to a final volume of 10 mL with deionized water. The absorbance of released ρ-nitrophenol was measured spectrophotometrically at 410 nm (UV-1601, Shimadzu Inc., Kyoto, Japan). All enzyme activity results are expressed as nmol h^−1^ g^−1^ dry soil. All the “Abbreviations” and “Its Definitions” were shown in Table 5.

#### 4.3.2. DNA Extraction and Sequencing Data Processing

Total genomic DNA was extracted from three replicate fresh soil samples (0.5 g each) using the PowerSoil^®^ DNA Isolation Kit (MoBio Laboratories, Carlsbad, CA, USA). DNA quality and quantity were determined using the Agarose gel electrophoresis and the Nanodrop1000 Spectrophotometer (Thermo Fisher Scientific, Waltham, MA, USA). The primer pairs 338F/806R (5′-ACTCCTACGGGAGGCAGCAG-3′/5′-GGACTACHVGGGTWTCTAAT-3′) and ITS1F/ ITS2 (5′-ACTTGGTCATTTAGAGGAAGTAA-3′/5′-BGCTGCGTTCTTCATCGATGC-3′) were used to amplify the V3–V4 region of bacterial 16S rRNA genes and ITS-1 region of fungal ITS genes, respectively [59,67].

Sequence processing was performed using QIIME, according to the following established criteria: (1) reads were truncated if the average quality score fell below 20 within a sliding 50 bp window and reads shorter than 50 bp were discarded; (2) reads were removed if they contained inexact barcode matches (no mismatches allowed), more than two primer mismatches or failed assembly; (3) sequence pairs were assembled using a minimum overlap of 10 bp. The processed sequences were then clustered into Operational Taxonomic Units (OTUs) at 97% similarity (3% dissimilarity) using USEARCH v5.2.32. The taxonomy of the 16S rRNA and fungal ITS-1 gene sequences within the OTUs was then assigned using the Quantitative Insights Into Microbial Ecology (QIIME) pipeline.

The abundances of soil total bacterial and fungal communities were used to represent the ratio of fungi-to-bacteria. Based on the relative abundance, the dominant microbial phyla were determined. In our study, the groups with relative abundances higher than 1% were identified, while those with less than 1% were grouped as “other”. The groups with significantly higher relative abundance than other microbial communities at the phylum level and with a cumulative relative abundance that accounted for more than 80% of the total were defined as the dominant microbial phyla. Sequencing data of soil bacteria and fungi have been deposited in the National Center for Biotechnology Information (NCBI) under accession numbers PRJNA1055189.

### 4.4. Calculation of Microbial Homeostasis, Threshold Element Ratios, and Carbon Use Efficiency

Soil stoichiometric imbalances between the available nutrients and microbial communities were the ratios of soil DOC:AN (DOC: AP or AN:AP) to MBC:MBN (MBC:MBP or MBN:MBP).The degree of microbial C:N, C:P, and N:P homeostasis (H′) can be calculated as follows [51]:

(1) H′=1/m

where m is the slope of the ln(DOC:AN, DOC:AP, or AN:AP) versus ln(MBC:MBN, MBC:MBP, or MBN:MBP) scatter plot. The H′ ≫ 1 indicates a strong stoichiometric homeostasis, with weak or no homeostasis when H′ ≈ 1 [45].

To explore the elements limiting metabolic activity, the threshold element ratios (TERs) of the resource C–nutrient ratio were calculated as follows [42]:

(2) $${TER}_{C:N}=\left({EA}_{C:N}\times {MB}_{C:N}\right)/n_{0}$$

(3) $${TER}_{C:P}=\left({EA}_{C:P}\times {MB}_{C:P}\right)/p_{0}$$

(4) $${EA}_{C:N}=BG/\left(LAP+NAG\right)$$

(5) $${EA}_{C:P}=BG/ACP$$

where MB_C:N_ denotes soil MBC:MBN ratio; MB_C:P_ denotes MBC:MBP ratio; the values of n_0_ and p_0_ represent the dimensionless normalized constants of N and P, respectively; n_0_ (p_0_) = e^intercept^, the intercept values of n_0_ and p_0_ are the standard principal axis regression between lnBG and ln(NAG + LAP) and lnBG versus lnACP, respectively.

Soil microbial CUE was calculated using the equation as follows [68,69]:

(6) $$CUE={CUE}_{max}\times {\left\{S_{C:N}\times S_{C:P}/\left[\left(K_{C:N}+S_{C:N}\right)\times (K_{C:P}+S_{C:P})\right]\right\}}^{0.5}$$

(7) $$S_{C:N}=\frac{{MB}_{C:N}}{L_{C:N}\times {EA}_{C:N}}$$

(8) $$S_{C:P}=\frac{{MB}_{C:P}}{L_{C:P}\times {EA}_{C:P}}$$

where the L_C:N_ and L_C:P_ represent the available soil nutrient ratios of soil DOC:AN and DOC:AP, respectively. K_C:N_ and K_C:P_ are half-saturation constants (0.5). CUE_max_, which indicates the upper limit of microbial growth efficiency was 0.6 [45,48].

### 4.5. Statistical Analysis

Two-way analysis of variance (ANOVA) was performed to test the effects of rhizodeposition and tree species on soil properties, stoichiometry, stoichiometric imbalances, TERs, microbial CUE, microbial diversity, and fungi-to-bacteria ratio. Relationship among soil stoichiometry, microbial property, and CUE was analyzed through Spearman correlation analysis. Statistical significance was defined at *p* < 0.05. Soil microbial alpha diversity was assessed using the Shannon–Wiener index, while beta diversity across the five forest ecosystems was visualized via non-metric multidimensional scaling (NMDS). Meanwhile, an investigation into the relationships between soil microbial communities and soil stoichiometry was conducted via redundancy analysis (RDA). Before RDA, the factor collinearity was tested in R with the VIF < 10. NMDS and RDA were performed in R Version 4.1.2 with the vegan package. Structural equation models (SEMs) were used to examine the key pathway of tree species regulating microbial CUE in the rhizosphere and bulk soils, respectively. In the SEM, the Principal Component Analysis (PCA) was used to reduce the number of variables to simplify our analyses and facilitate interpretations, including soil available nutrient stoichiometry (DOC:AN, DOC:AP, and AN:AP), soil enzyme stoichiometry (BG:(NAG + LAP), BG:AP, and (NAG + LAP):AP), microbial biomass stoichiometry (MBC:MBN, MBC:MBP, and MBN:MBP) and soil microbial composition (bacterial and fungal). The scores of the composite variables were based on the first dimensions of the PCA biplots. The SEM analyses were conducted using AMOS 20.0 software (Amos Development Corporation, Chicago, IL, USA).

## 5. Conclusions

We highlighted the differences in soil stoichiometry and microbial CUE between rhizosphere and bulk soil, as well as among the tree species. Soil C, N, and P and their stoichiometry, microbial community composition, and microbial CUE were significantly different between rhizosphere and bulk soils among all tree species. The quality of soil nutrient availability and the composition of microbial communities are closely related to microbial CUE. Moreover, our results demonstrated that the tree species can change the soil microbial community and soil stoichiometry, potentially altering microbial CUE. Notably, compared with rhizosphere soil, microbial CUE in bulk soil was more sensitive to variations in available nutrients and microbial biomass stoichiometry. Altogether, our data provide an important perspective linking soil microbial communities to soil stoichiometry and microbial CUE in the rhizosphere and bulk soil among different tree species. These results are direct evidence of the relationship between vegetation composition, microbial characteristics, and soil carbon cycling in important ecological functional areas.

## Figures and Tables

**Table 1:** Soil C, N, P, available nutrients, microbial biomass, and enzymatic activities of rhizosphere (RS) and bulk soil (BS) among different tree species.

Soil Parameters	Soil Positions	*B. platyphylla*	*P. davidiana*	*F. mandschurica*	*L. gmelinii*	*P. koraiensis*
DOC (mg^.^kg^−1^)	RS	314.0 ± 14.8 Aa	255.5 ± 6.9 Ab	266.2 ± 8.4 Ab	194.1 ± 8.0 Ac	296.6 ± 13.7 Aa
	BS	140.4 ± 4.9 Bc	153.3 ± 4.7 Bb	140.0 ± 3.6 Bc	107.3 ± 4.9 Bd	188.6 ± 10.3 Ba
AN (mg^.^kg^−1^)	RS	114.6 ± 6.3 Aa	98.1 ± 5.1 Abc	102.0 ± 3.4 Ab	62.4 ± 2.0 Ad	91.1 ± 6.8 Ac
	BS	47.3 ± 4.3 Bab	42.9 ± 5.1 Bb	32.1 ± 2.2 Bc	25.8 ± 1.5 Bd	50.4 ± 2.5 Ba
AP (mg^.^kg^−1^)	RS	5.5 ± 0.5 Ad	5.1 ± 0.6 Ad	12.1 ± 0.7 Ac	48.1 ± 1.0 Ab	55.5 ± 2.6 Aa
	BS	2.2 ± 0.2 Bd	1.7 ± 0.1 Be	3.2 ± 0.1 Bc	8.7 ± 0.6 Bb	26.2 ± 1.1 Ba
MBC (mg^.^kg^−1^)	RS	1546.1 ± 55.2 Aa	1300.6 ± 50.8 Ab	633.1 ± 32.8 Ad	536.8 ± 10.6 Ae	922.6 ± 37.1 Ac
	BS	1376.0 ± 26.2 Ba	654.4 ± 11.7 Bb	532.0 ± 21.6 Bc	449.7 ± 20.3 Be	491.7 ± 19.4 Bd
MBN (mg^.^kg^−1^)	RS	244.4 ± 9.7 Aa	252.1 ± 16.5 Aa	94.9 ± 10.3 Ac	60.7 ± 5.5 Ad	156.9 ± 6.7 Ab
	BS	182.6 ± 5.4 Ba	90.4 ± 3.0 Bb	77.7 ± 5.5 Bc	55.3 ± 3.5 Bd	73.1 ± 6.2 Bc
MBP (mg^.^kg^−1^)	RS	54.9 ± 1.6 Aa	52.8 ± 1.0 Aa	39.6 ± 1.2 Ab	25.6 ± 0.8 Ac	24.1 ± 0.9 Ac
	BS	43.2 ± 1.7 Ba	36.0 ± 2.4 Bb	36.5 ± 1.3 Bb	23.0 ± 1.0 Bc	21.6 ± 0.6 Bc
BG (nmol^.^g^−1.^h^−1^)	RS	1601.6 ± 32.7 Aa	1641.8 ± 32.6 Aa	1349.5 ± 22.6 Ab	1232.7 ± 23.3 Ac	1270.2 ± 11.8 Ac
	BS	1295.1 ± 26.5 Ba	441.7 ± 8.7 Be	602.0 ± 17.2 Bc	525.5 ± 13.7 Bd	1066.2 ± 24.9 Bb
NAG + LAP (nmol^.^g^−1.^h^−1^)	RS	583.3 ± 13.9 Aa	440.9 ± 15.4 Ab	423.3 ± 10.4 Ab	435.8 ± 9.7 Ab	257.1 ± 6.2 Ac
	BS	450.4 ± 7.5 Ba	133.7 ± 9.0 Be	146.4 ± 3.3 Bd	180.6 ± 7.8 Bc	251.4 ± 4.0 Ab
ACP (nmol^.^g^−1.^h^−1^)	RS	348.1 ± 4.6 Ad	375.6 ± 7.6 Ac	377.7 ± 3.4 Abc	387.6 ± 6.5 Ab	426.3 ± 6.7 Aa
	BS	267.1 ± 7.5 Bc	282.0 ± 1.1 Bb	171.9 ± 2.4 Be	245.7 ± 1.4 Bd	400.4 ± 2.8 Ba
SOC	RS	93.2 ± 7.3 Ab	95.4 ± 6.0 Ab	72.4 ± 6.4 Ac	56.2 ± 4.8 Ad	129.5 ± 9.0 Aa
(g^.^kg^−1^)	BS	40.7 ± 3.6 Bb	23.8 ± 2.2 Bd	29.2 ± 1.9 Bc	24.3 ± 3.5 Bcd	68.4 ± 5.8 Ba
TN	RS	6.2 ± 0.5 Aa	6.0 ± 0.3 Aa	5.6 ± 0.2 Ab	3.3 ± 0.2 Ac	6.7 ± 0.5 Aa
(g^.^kg^−1^)	BS	2.9 ± 0.2 Bb	2.0 ± 0.1 Bc	2.2 ± 0.1 Bc	2.2 ± 0.1 Bc	5.3 ± 0.2 Ba
TP	RS	0.6 ± 0.0 Ac	0.57 ± 0.0 Ac	0.8 ± 0.0 Ab	0.8 ± 0.0 Ab	1.3 ± 0.1 Aa
(g^.^kg^−1^)	BS	0.5 ± 0.0 Bc	0.3 ± 0.0 Be	0.4 ± 0.0 Bd	0.6 ± 0.0 Bb	0.8 ± 0.00 Ba

Note: Different uppercase letters indicate significant differences between the rhizosphere and bulk soil (*p* < 0.05); different lowercase letters indicate significant differences among the tree species (*p* < 0.05). DOC, dissolved organic C; AN, available nitrogen; AP, available phosphorus; MBC, microbial biomass carbon; MBN, microbial biomass nitrogen; MBP, microbial biomass phosphorus; BG, β-1,4-glucosidase; NAG, β-1,4-N-acetylglucosaminidase; LAP, leucine aminopeptidase; ACP, acid phosphatase; SOC, soil organic carbon; TN, total nitrogen; TP, soil total phosphorus.

**Table 2:** Microbial CUE of rhizosphere (RS) and bulk soil (BS) among different tree species.

Soil Parameters	Soil Positions	*B. platyphylla*	*P. davidiana*	*F. mandschurica*	*L. gmelinii*	*P. koraiensis*
Microbial CUE	RS	0.22 ± 0.01 Ad	0.20 ± 0.01 Ad	0.25 ± 0.02 Ac	0.43 ± 0.01 Aa	0.36 ± 0.01 Ab
	BS	0.19 ± 0.01 Bc	0.18 ± 0.01 Bd	0.16 ± 0.01 Be	0.35 ± 0.01 Ba	0.33 ± 0.01 Ba

Note: Different uppercase letters indicate significant differences between the rhizosphere (RS) and bulk soil (BS) (*p* < 0.05); different lowercase letters indicate significant differences among the tree species (*p* < 0.05).

**Table 3:** Variations in soil microbial alpha diversity (Shannon diversity index) and fungi-to-bacteria ratio along the different tree species.

Soil Parameters	Soil Positions	*B. platyphylla*	*P. davidiana*	*F. mandschurica*	*L. gmelinii*	*P. koraiensis*
Bacterial Shannon index	RS	7.25 ± 0.20 Aa	6.94 ± 0.12 Ab	7.30 ± 0.05 Aa	7.19 ± 0.07 Aa	7.13 ± 0.02 Ab
	BS	6.88 ± 0.04 Bb	6.79 ± 0.07 Bc	6.63 ± 0.02 Bc	7.05 ± 0.03 Ba	6.88 ± 0.05 Bb
Fungal Shannon index	RS	4.77 ± 0.20 Aa	4.36 ± 0.59 Ab	4.07 ± 0.22 Ab	4.74 ± 0.07 Aa	4.78 ± 0.05 Aa
	BS	4.26 ± 0.07 Ba	3.28 ± 0.04 Bd	3.89 ± 0.13 Bc	4.37 ± 0.04 Ba	4.12 ± 0.03 Bb
Fungi-to-bacteria ratio	RS	0.98 ± 0.07 Ab	1.04 ± 0.07 Aab	1.03 ± 0.03 Bab	1.10 ± 0.06 Ba	1.09 ± 0.01 Aa
	BS	1.05 ± 0.08 Ab	0.99 ± 0.04 Ab	1.14 ± 0.02 Aa	1.19 ± 0.03 Aa	0.99 ± 0.01 Bb

Note: Different uppercase letters indicate significant differences between the rhizosphere (RS) and bulk soil (BS) (*p* < 0.05); different lowercase letters indicate significant differences among the tree species (*p* < 0.05).

**Table 4:** Site basic characteristics of the 0–10 cm layer of the five forest types.

Forest Types	*B. platyphylla*	*P. davidiana*	*F. mandschurica*	*L. gmelinii*	*P. koraiensis*
Stand age	53	55	47	50	58
Elevation (m)	305 ± 5	307 ± 3	305 ± 4	314 ± 4	307 ± 2
DBH (cm)	15.30 ± 5.41	12.78 ± 4.03	12.34 ± 3.2	14.48 ± 4.40	26.66 ± 4.75
Tree Height (m)	10.58 ± 0.53	9.60 ± 1.04	9.97 ± 0.84	11.42 ± 1.05	10.80 ± 1.12
Soil pH	6.06 ± 0.10	6.10 ± 0.05	6.35 ± 0.11	6.00 ± 0.04	6.20 ± 0.20
Soil bulk density (mg^.^kg^−3^)	1.09 ± 0.20	1.13 ± 0.20	0.93 ± 0.27	0.98 ± 0.04	0.87 ± 0.11

Note: DBH, diameter at breast height.

**Table 5:** The abbreviations and their definitions in our study.

Definitions	Abbreviations
Carbon	C
Rhizosphere soil	RS
Bulk soil	BS
Microbial carbon use efficiency	CUE
*Betula platyphylla* Suk. forest	*B. platyphylla*
*Fraxinus mandschurica* Rupr. forest	*F. mandschurica*
*Populus davidiana* Dode. forest	*P. davidiana*
*Larix gmelinii* (Rupr.) Kuzen. forest	*L. gmelinii*
*Pinus koraiensis Siebold et* Zucc. forest	*P. koraiensis*
the average diameter at breast height	DBH
Soil water content	SWC
Soil organic carbon	SOC
Soil total nitrogen	TN
Soil total phosphorus	TP
Dissolved organic carbon	DOC
Dissolved organic nitrogen	DON
Soil available nitrogen	AN
Soil available phosphorus	AP
Soil microbial biomass carbon,	MBC
Soil microbial biomass nitrogen	MBN
Soil microbial biomass phosphorus	MBP
β-1,4-glucosidase	BG
β-1,4-N-acetylglucosaminidase	NAG
leucine aminopeptidase	LAP
Acid phosphatase	ACP
The threshold element ratios	TERs
Two-way analysis of variance	ANOVA
Non-metric multidimensional scaling	NMDS
Redundancy analysis	RDA
Structural equation models	SEM
Principal Component Analysis	PCA

## Data Availability

The sequencing data of soil bacteria and fungi have been deposited in the National Center for Biotechnology Information (NCBI) under accession numbers PRJNA1055189.

## Supplementary Materials

The following supporting information can be downloaded at: https://www.mdpi.com/article/10.3390/plants15040652/s1, Table S1: Results of the two-way repeated measures ANOVA concerning the effects of soil positions, tree species, and their interaction on the soil available nutrient stoichiometry, microbial biomass stoichiometry, and enzyme stoichiometry; Table S2: Pearson correlation coefficients between soil stoichiometry, microbial carbon use efficiency and microbial diversity.
