# Supplementary material for: Soil Stoichiometry-Regulated Microbial Carbon Use Efficiency Between Rhizosphere and Bulk Soils in the Temperate Forests of Northeastern China
Source: Plants (Basel). 2026 Feb 20;15(4):652. doi: 10.3390/plants15040652 (PMC12944563; doi:10.3390/plants15040652)
Supplement: Supplementary file 1 [file plants-15-00652-s001.zip › Supplementary_Material/TableS1.pdf]

Supplementary Material

Table S1 Results of the two-way repeated measures ANOVA concerning the effects of soil positions, tree species, and their interaction on the soil available nutrient stoichiometry, microbial biomass stoichiometry, and enzyme stoichiometry.

**Soil Parameters**	**Soil positions**	**Tree species**	**Soil positions×Tree species**
DOC:AN	1.000	< 0.001	1.000
DOC:AP	< 0.001	< 0.001	< 0.001
AN:AP	< 0.001	< 0.001	< 0.001
MBC:MBN	< 0.001	< 0.001	< 0.001
MBC:MBP	< 0.001	< 0.001	< 0.001
MBN:MBP	< 0.001	< 0.001	< 0.001
BG: (NAG+LAP)	< 0.001	< 0.001	< 0.001
BG:ACP	0.884	< 0.001	< 0.001
(NAG+LAP) :ACP	< 0.001	< 0.001	< 0.001
C:N imbalance	< 0.001	< 0.001	< 0.001
C:P imbalance	< 0.001	< 0.001	< 0.001
N:P imbalance	< 0.001	< 0.001	< 0.001
TERC:N	< 0.001	< 0.001	< 0.001
TERC:P	< 0.001	< 0.001	< 0.001
CUE	0.001	< 0.001	0.003
